# Studying social workers’ roles in natural disasters during a global pandemic: What can we learn?

**DOI:** 10.1177/1473325020973449

**Published:** 2021-03

**Authors:** Claire Antonia Crawford

**Affiliations:** Texas Children's Hospital, University of Texas Health Science Center at Houston, USA

**Keywords:** Disasters, emergency social work, social justice, health, social work practice, participant observation

## Abstract

The author reflects on the convergence of her roles as a qualitative researcher studying social workers’ roles during Hurricane Harvey, a student of public health, and a hospital social worker in the midst of the COVID-19 pandemic. Similarities are drawn between the social work role following a natural disaster and a pandemic disaster along with observations regarding core differences. Practice and research recommendations are provided for social workers in the domains of therapeutic interactions, social justice, and public health. While therapeutic relationships have often been far more difficult to achieve during the pandemic than Hurricane Harvey, the assistance of technology and proper personal protective equipment has been helpful in filling communication gaps. Both types of disasters are universal in their reach, impacting people of all backgrounds; the social work role has been to address differences in access to resources, including health care and financial assistance. Finally, social workers play a significant role in public health during disasters through disseminating reliable information about safety, resources, and opportunities to assist others. The author recommends the expansion of social work in the public health space to provide more insight about communicating with vulnerable populations during disasters.

## Introduction

My worlds as a student, a researcher, and a social worker collided on April 7, 2020. After four years of coursework, interviews, analysis, and writing, I would finally defend my dissertation to complete my degree in public health. Due to the COVID-19 pandemic, I presented a closed dissertation defense to my committee on WebEx was greeted with a joyful chorus of “Congratulations, Dr. Crawford!” at my kitchen table. When I shut my computer and started to realize that the culmination of not only four years of work but also a lifetime of dreaming about getting my PhD had come to an end, I was surprised to find myself with a greater sense of curiosity than completion.

Before I knew it would become the topic of my dissertation and years of study, Hurricane Harvey brought rain, floods, displacement, illness, injury, and death to my city Houston in 2017. I decided to explore the efforts of social workers who responded to the many people affected by Harvey for my dissertation, interviewing 39 participants who worked at a local children’s hospital or volunteered at public shelters in the area. As I interviewed social workers working in and around Houston, we discussed their roles at shelters or the hospital, what Houston’s residents seemed to need the most, who was at highest risk for severe stressors in Harvey’s aftermath, what kind of support was needed and when and, most importantly, what they hoped would be better the next time we faced a major disaster.

I had a first-hand view as one of the 15 social workers who participated in “riding out” the storm at Texas Children’s Hospital, as reflected from my participant-observer status in the study. I have since been working as a palliative care social worker at Texas Children’s Hospital, where I witness every day the impact of COVID-19 on children, families, and employees.

## Comparing disasters

As I began to ponder our previous disaster and the current pandemic, I wondered what social workers could learn from natural disasters like hurricanes and where our roles would diverge. I wasn’t alone: colleagues of mine who knew of my research started asking questions like, “Ok, you’re our disaster researcher, so what should social workers be doing now?” I felt frustrated and helpless when I realized that I had far more questions than answers. We seemed, as an entire society, more defined by our limitations than our abilities during COVID-19. Below are several of the observations and anecdotal evidence I have gathered regarding overlap between social workers’ roles in two very different disaster settings after three years of natural disaster research, four months of pandemic social work practice, and a lifetime of curiosity as a student:

### Therapeutic interactions

The first thing I noticed working during COVID-19 is that the way social workers interact with patients and families has, necessarily, changed drastically. During and after Harvey, personal interactions were essential for providing emotional support. In particular, social workers cited listening to others as the most common intervention in the shelter setting, saying that this simple act acknowledged and validated the traumatizing experience of surviving a disaster. Therefore, listening to others was at the forefront of my mind during COVID-19, and I wanted to provide the comforting presence for hospitalized patients and families that could bring peace if only temporarily.

I quickly found that therapeutic interactions were much harder to achieve during a pandemic. First, social workers had very limited face-to-face interactions with families per hospital policy to reduce the spread of COVID-19. This policy resulted in many a phone call ending in a voicemail from a social worker, offering to talk over the phone to meet needs and provide support. Personally, I rarely reached a family member during the phone call or received a call back. On the occasions when I did reach a family member, even one with whom I had previously established a strong in-person relationship, our calls were exceptionally brief and generally superficial. The listening social workers relied on so heavily in previous disasters was no longer available in the same way, and I felt hard-earned therapeutic alliances begin to disintegrate.

When I was able to meet with a family in person, usually in end-of-life circumstances, I could not reach out to lay a hand on a mother’s shoulder as tears dropped into her lap or embrace a father after he said goodbye to his daughter for the last time. The physical distance felt like an exponentially larger emotional gulf.

With time, patience, and practice, we hospital social workers have figured a few things out. A steadier supply of personal protective equipment has allowed us to venture into more patients’ rooms, signaling a return to much of the therapeutic alliances we have made (though I have learned that foggy goggles are not conducive to meaningful eye contact). When restrictions are added due to a potential COVID-19 exposure or pending tests, we palliative social workers stay away but give our physicians who are still able to enter the rooms an informative pamphlet with information about how we can help. We have added a few resources, like deep breathing exercises and meditation apps, to assist in our absence. Finally, the often avoided telehealth has seen a surge that seems unlikely to go away any time soon. We have been able to participate in some of these appointments with the added benefit of video communication to enhance the experience on both sides of the camera.

Hurricane Harvey taught us that listening and therapeutic presence is essential to carry others through the emotional trauma of a disaster. The pandemic has taught us that this therapeutic presence is indeed a necessity in our role as social workers and that adapting our practice is essential. Whether we are communicating on the phone, video, or in person, social workers must find ways to relay empathy and validate the experiences of our clients to demonstrate that they are not emotionally isolated, even when they are physically alone. In the likely even that natural disasters occur during the pandemic, providing therapeutic listening should remain a priority for volunteer social workers through whichever communication media are available.

### Social justice

One component of any type of disaster that fascinates me is the indiscriminate direct impact; in the pandemic as in Harvey, everyone is subject to detrimental effects. For example, wealthy and poor neighborhoods alike flooded, people across the city had to be rescued, and many were faced with completely rebuilding their homes. During the pandemic, people across the entire world have fallen ill, often fatally, with no single group being completely exempt. Interestingly, in both cases, older adults were among the most vulnerable to serious long-term impacts, including severe illness and death.

The difference between the haves and have-nots in both cases seems to be access to resources. Despite the ubiquitous nature of COVID-19, the ability of the sick to receive the healthcare they need (i.e. acute hospital care and ventilators when needed) has become a true determinant of life and death (King, 2020). After Hurricane Harvey, people with stable jobs could continue to receive income; those who could and knew to pay for flood insurance were at least partially covered; those who had a stable and well-resourced social network had a place to stay when their homes were uninhabitable; those with significant savings could afford to return to comfortable homes once repaired.

The role of social workers to provide to those in most need has been integral during both events. During Harvey, people who resorted to public shelters for temporary housing needed both short-term accommodations (food, lodging, medications, clothing, diapers, etc.) and long-term resource referrals (Federal Emergency Management Agency [FEMA], unemployment, and legal aid). People returning to their homes needed physical presence of others to assist with mucking and gutting their homes before they could consider hiring professional contractors to begin assessing and rebuilding. Many social workers heeded calls for help and stepped in to provide what is considered more traditional social work, such as referring to reliable resources, directing to appropriate agencies and volunteering groups, and as spontaneous volunteers who mucked and gutted houses and gathered donations.

Arguably, the pandemic has resulted in longer-term assistance needs with the caveat that no one knows how long the pandemic’s effects could last. Unemployment has reached a historic high at 14.7% in April (U.S. Bureau of Labor Statistics, 2020), resulting in profound difficulties paying rent, mortgages, or health care related to COVID-19 itself as many people depend on their employers for health insurance (King, 2020). Confusion regarding policies on the local, state, and national levels have left workers confused about their employment status, and $1,200 stimulus checks for some have barely made a dent in meeting needs. Many social workers may find themselves in the large group facing unemployment, pay cuts, or, like me, mandatory reduced time at work. Of course, once a person contracts COVID-19, variation in insurance could result in unexpectedly large bills for hospital stays or testing while sick time at work quickly dissolves. While there was an easy distinction during Harvey between the social workers whose homes or lives were disrupted and those who remained relatively unphased, it is difficult to find anyone who has not lost something or someone during the pandemic. The truly universal nature of COVID-19 has left social workers with less emotional and financial capability to be helpful outside of a work setting.

Perhaps the takeaway regarding social workers actively participating in social justice health initiatives is that our work should be more proactive than reactive. If we know far before disasters of any kind occur that there are profound disparities in access to health care, for example, then we can assume that those disparities will be magnified as disasters disproportionately affect those who already have fewer resources. We should be pursuing social justice before and during disasters, taking an upstream approach.

I would consider one of the greatest accomplishments for social justice during COVID-19 to be the nation-wide protests to bring justice for George Floyd, a Black man killed by the police in Minnesota on May 25, 2020. Following the peaceful protests, four men were charged for Floyd’s murder (CBS Minnesota, 2020), and a nationwide conversation about systemic racism was opened more than ever before. For myself and many others, questions about the safety of participation in these protests loomed; if both systemic racism and COVID-19 are public health crises, how can we effectively respond to both? Wearing masks and distancing as much as possible from others, social workers joined in across the country to chant, “Black Lives Matter, and “No justice, no peace.” Recognizing that there is still a long way to go to obtain justice for other victims of police brutality, including Breonna Taylor, social workers and many, many activists have successfully disrupted racism and white supremacy despite the challenges of a global pandemic. Protesters in this pandemic has demonstrated that we can tackle justice for multiple advocacy efforts at once and that, importantly, our work for one cause does not pause when another arises. We can and should hold the truth that systemic racism and disasters may co-occur, each amplifying the detrimental effects of the other.

### Public health

Perhaps the area in which social workers’ roles overlap the most between the natural disaster and pandemic is through the dissemination of reliable information, often through social media. During my research, I was fascinated by how many participants used social media platforms (particularly Facebook and Twitter) to provide reliable, helpful information to others about both assisting others and receiving assistance. They would repost and write status updates about where and how to volunteer time, what to do when volunteering, what items were needed for donations, where donation drives were taking place, how to stay safe during the storm, how to return children to school, and so much more. When they were unable to leave their homes, they looked for ways to serve from a distance.

During this pandemic, many social workers have made the choice to stay home not because of physical barriers like floodwater, but due to a mixture of anxiety about contracting the disease and fear of unknowingly passing COVID-19 to others. Misinformation abounds in the digital age, but many social work colleagues of mine from every type of work setting have once again utilized social media to share public health data, informed others about safety techniques such as proper hand hygiene and mask wearing, and reinforced new concepts such as social distancing and flattening the curve. While we have learned that social workers have a place in disseminating information about resources during natural disasters, COVID-19 has taught us that information-sharing is not always straightforward. Rather, in this time of widespread misinformation and politicization of public health, there has been a greater need for identifying reliable sources and correcting misconceptions. Social workers should prepare for the event that future natural disasters could follow this misinformation trend and assume that local governments may be met with skepticism in preparing for and responding to disasters.

## Conclusion

In this article, the author has compiled findings from her qualitative doctoral dissertation on social workers’ roles during a natural disaster to compare them with anecdotal observations, conversations with colleagues, and critical reflections about practicing social work during COVID-19. Specifically, similarities and differences between the two types of disasters were drawn in the domains of therapeutic relationships, social justice, and public health.

While therapeutic relationships were one of the most important things social workers did after Harvey, they are significantly and often insurmountably harder during a pandemic in which physically separating ourselves from others is the best way to help them. In both disasters, differences in access to healthcare have played a large role in long-term impacts, including likelihood of severe illness and death. Social workers have a responsibility to help increase access to health care for all who need it while continuing to fight for social justice in all realms of public safety. Alongside others, many social workers participated in nationwide protests during the pandemic to call for an end to systemic racism.

Social workers have a larger role to play in public health than we often consider, and I would argue that this role should be expanded. Alongside other helping professionals such as doctors, nurses, and public health practitioners, social workers have been integral in both disasters in distributing safe, reliable information to those who might not otherwise be aware of important safety measures. In response to recent politicization of basic public health mandates and announcements, social workers should also be prepared to correct misconceptions. Due to social workers’ awareness of systems, human behavior, and available resources, more social workers should be included in the public health space to reach populations that are most vulnerable during disasters.
